# Characterization of Mild Delayed Gestational Hypertension in Rats Following Ozone Exposure

**DOI:** 10.21203/rs.3.rs-3977101/v1

**Published:** 2024-02-23

**Authors:** Russell Hunter, Thomas Wilson, Selita Lucas, David Scieszka, Barry Bleske, Andrew Ottens, Ryan Ashley, Carolyn Pace, Nancy Kanagy, Matthew J Campen

**Affiliations:** University of New Mexico College of Pharmacy; University of New Mexico College of Pharmacy; University of New Mexico College of Pharmacy; University of New Mexico College of Pharmacy; University of New Mexico College of Pharmacy; Virginia Commonwealth University; New Mexico State University; University of New Mexico School of Medicine; University of New Mexico School of Medicine; University of New Mexico College of Pharmacy

**Keywords:** Ozone, Air Pollution, Pregnancy, Hypertension

## Abstract

The contribution of air pollution induced cardio-pulmonary damage on the development of hypertensive disorders of pregnancy and other adverse outcomes of pregnancy has gained increased attention as epidemiological data continues to highlight spatiotemporal pregnancy trends related to air pollution exposure. However clinical mechanistic data surrounding gestational complications remains sparse, necessitating the need for the use of animal models to study these types of complications of pregnancy. The current study seeks to examine the real-time effects of mid-gestational ozone exposure on maternal blood pressure and body temperature through the use of radiotelemetry in a rat model. The exposure resulted in acute depression of heart rate and core body temperature as compared to control animals. Ozone exposed animals also presented with a slight but significant increase in arterial blood pressure which was perpetuated until term. The data presented here illustrates the feasibility of murine models to assess cardiovascular complications caused by inhaled toxicants during the window of pregnancy.

## Introduction

Air pollution has a known impact on vascular function, driving an increased incidence of inflammatory vascular diseases and hypertension [1]. Recent studies have identified a potential link between ambient air pollution and incidence of gestational hypertension or preeclampsia, showing positive associations with ambient ozone (O_3_), nitrogen dioxide, and particulate matter [2–5]. Importantly, using time-series statistical analyses, epidemiological studies highlight that the vulnerable window for this relationship appears to be in early pregnancy [5, 6], potentially during placentation and early development of the placenta. These associations remain incompletely characterized in terms of biological plausibility and pathogenesis, with very few toxicological studies conducted to specifically address pollutant-induced gestational hypertension [7]. Notably, epidemiologically-observed blood pressure changes in late-stage pregnancy have been most clearly aligned with first trimester exposures to air pollution [8].

Controlled exposures to O_3_ promote uterine and placental vascular responses in preclinical models and help explain the biological plausibility of epidemiological assosications. The Kodavanti lab identified uterine artery pathophysiological changes associated with early gestational exposure to 0.4 and 0.8 ppm O_3_. Exposure at implantation (GD5.5) led to small but significant increases in the uterine artery resistance and pulsatile indices at GD21 [7]. In recent studies of inhaled O_3_ in pregnant rats, we have observed substantial serum elevations of soluble Fms-like tyrosine kinase-1 (aka sFlt-1) [9], which is an essential player in both preeclampsia and peripartum cardiomyopathy [10–12]. Proteomic and phosphoproteomic changes in the cardiac tissue, typified by cardiomyopathic ontological classifications, from pregnant rats exposed to O_3_ at GD10.5 were dramatically greater than the changes seen in dams exposed at GD20.5, suggesting that an early impact on placental growth and development may be central to late stage changes.[9] Furthermore, we have observed significant alterations of uterine vascular physiology and placental transcription products with late-stage O_3_ exposure[13]. Thus, recent preclinical research does indicate a path to gestational hypertension from early- or mid-gestational exposure to air pollution.

A missing component of the preclinical investigations into early gestational impacts of air pollution exposure on maternal cardiovascular health is a clear indicator of systemic hypertension, or blood pressure change, especially as develops in late pregnancy and resolves upon delivery (and removal of the offending placenta). In the present study, we therefore sought to assess O_3_ exposure effects on blood pressure and related parameters using radiotelemetry for continuous measurement with minimal handling of the pregnant rats.

## Materials and Methods

### Animals

Female Sprague-Dawley rats were used in this study. Animals were housed in an Association for Assessment and Accreditation of Laboratory Animal Care (AAALAC)-approved facility at the University of New Mexico Health Sciences Center and maintained at recommended temperature (20–24°C), relative humidity (30–60%), and on a 12-h light/dark cycle throughout the study. Animals were provided with standard chow and water ad libitum. All animal procedures were conducted humanely and approved by the Institutional Animal Care and Use Committee at the University of New Mexico.

### Radiotelemetry Implantation

Rats were implanted with radiotelemetry devices for continuous acquisition of systemic arterial blood pressure, heart rate (HR), core body temperature (T_CO_), and physical activity (PA; HD-S10; DataSciences, Intl; St Paul, MN). Implantation was conducted as previously described. Briefly, under isoflurane anesthesia, the pressure catheter of the telemetry device was advanced through the insertion point of the femoral artery to a position within the abdominal aorta and secured with suture and tissue glue. The body of the telemeter was secured subcutaneously in the flank of the rat. Buprenorphine (0.03 mg/kg subcutaneous) was administered 30 min before the end of surgery and postoperatively as needed to provide analgesia. Rats recovered a minimum of 7 days prior to further experimentation.

### Mating

**Figure 1:**
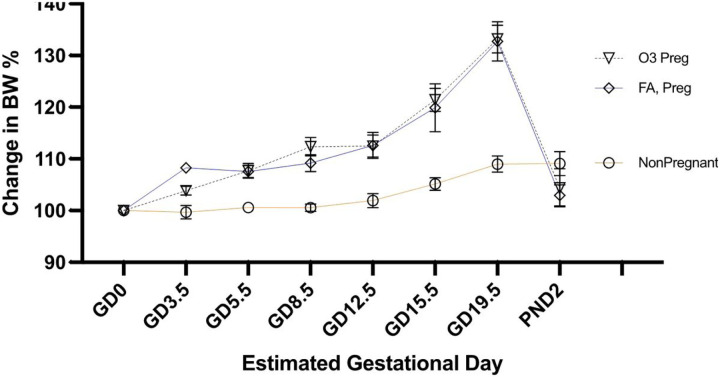
Normalized body weight trends for telemetered rats (third experiment shown) following pairing with male rats. By GD8.5 sufficient discrimination was evident to select and randomize rats for exposures. No differences were seen with O_3_ exposure

Once rats recovered from surgery, the fidelity of the blood pressure signal was confirmed (a minimum pulse pressure of 10 mmHg was required). Rats with viable blood pressure signals were then paired with male rats for up to 3 days, with confirmation of successful copulation as evidenced bu the presences of a seminal plug. Following successful plug check or after three days of pairing, female rats were removed and returned to normal housing conditions. Body weights were assessed at regular intervals following this pairing. A > 5% increase in body weights at GD8.5 was consistent with pregnancy (Fig. 1). Pregnant rats were then exposed to O3 at mid gestation on GD10.5 (described below). Non-pregnant rats were considered for re-pairing, assuming the radiotelemetry signal remained strong. Three experiments were undertaken to obtain an N = 7 pregnant rats per exposure group with radiotelemetry signals of sufficient quality. Following exposures, rats were followed to term and delivery.

### O_3_ Exposure

Dams were exposed to either filtered air (FA) or 1ppm O_3_ for a single 4-h period via whole body inhalation. O_3_ was generated using an OREC silent arc discharge generator (Osmonics, Phoenix, Arizona) as previously described [9, 14]. Water was available for animals during the exposure, but food was withheld. Exposures took place in standard shoebox cages without bedding (to prevent O_3_ scavenging). O_3_ concentrations were monitored in real-time using an indoor air quality monitor (GrayWolf Sensing Solutions, Shelton, CT) and adjustments to flow were conducted manually to ensure that concentrations were consistently between 0.9–1.1 ppm.

### Data Analysis and Statistics

Data from all rats with sufficient signal quality was included (namely, pulse pressure > 10 mmHg). Data for mean arterial blood pressure (MAP), HR, T_CO_, and PA were collected every 10 minutes, but averaged to daily values for statistical testing. FA and O_3_ groups were compared by a 2-way repeated measures ANOVA to account for time and exposure, with Šídák’s multiple comparisons test used to confirm O3 effects at specific time points (GraphPad Prism, v9.5.1). Data are presented as mean +/− SEM; p-values < 0.05 were considered to be significant.

## Results and Conclusions:

### Animal Weights

Animals were weighed weekly to assess differences in weight gain and as a measure of overall health. Following pairing, female rats were weighed every 2–4 days to assess likelihood of pregnancy (Fig. 1). Growth greater than 5% at GD8.5 allowed for a clear demarcation of pregnancy, at which dams were randomized for exposure conditions (FA or O_3_).

### Mean arterial blood pressure

**Figure 2:**
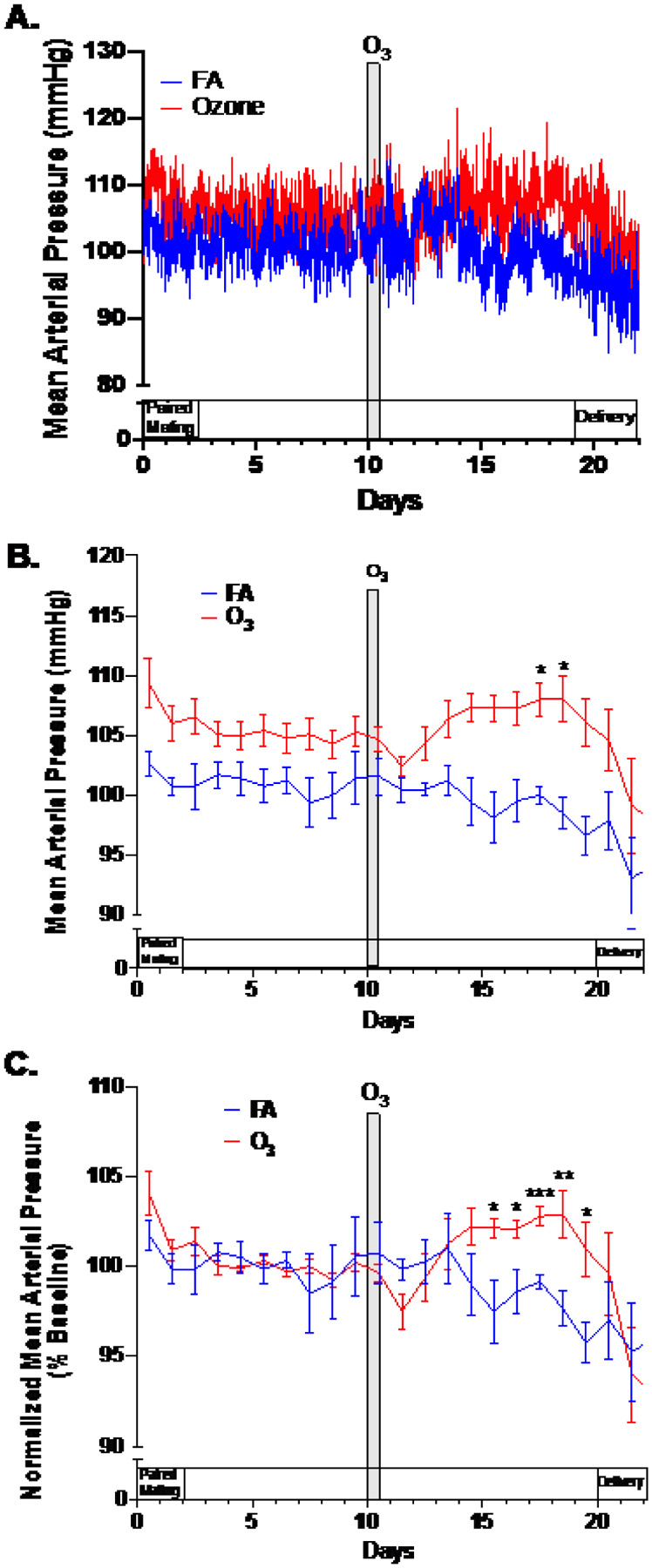
Mean arterial blood pressure from pregnant rats (n=7/grp) exposed to filtered air (control) or 1 ppm O_3_ for 4h on GD10.5. Shown are (A) raw data (10 minute collections, averaged by group); (B) 24h average data; and (C) data normalized to average readings from GD3.5–GD8.5, a period where handling was minimized and pairing with male rats was complete. Data normalization in (C) was conducted to offset initial group differences apparent in the raw data (A and B). Asterisks indicate significant difference between the FA and O_3_ groups, based on a 2-way repeated measures ANOVA with Šídák’s post-hoc testing (*p<0.05; **p<0.01; ***p<0.001) using GraphPad Prism

Evidence of a late-gestational increased in blood pressure was determined in O_3_-exposed rats (Fig. 2). Prior to exposures, the FA and O_3_ groups, despite randomization, displayed an approximately 3.5 mmHg difference in mean arterial pressure, possibly real or due to calibration variance in the radiotelemetry devices. Regardless, following O_3_ exposure at GD10.5, this difference became augmented by approximately 5.0 mmHg from approximately GD15.5 until delivery. To better ascertain the time frame of the increased mean arterial pressure, we normalized values to the period from GD3.5 to GD8.5, when paired mating was over but prior to exposures. With this approach, we revealed significant differences between FA and O_3_-exposed groups from GD15.5 until GD19.5. Notably, deliveries began between GD20.5 and GD22.5, at which point mean arterial pressure differences between exposure groups subsided.

### PA, HR, and T_CO_

**Figure 3:**
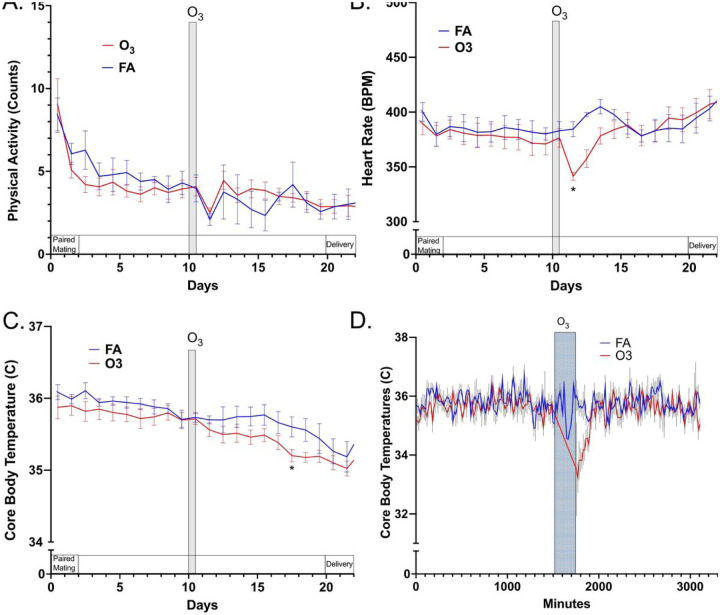
Physical activity (A), heart rate (B), core body temperature (C) for the full 22 day period of pregnancy, and more detailed resolution of core body temperature relative to exposure (D) from pregnant rats (n=7/grp) exposed to filtered air (control) or 1 ppm O_3_ for 4h on GD10.5. Asterisks indicate significant difference between the FA and O_3_ groups, based on a 2-way repeated measures ANOVA with Šídák’s post-hoc testing (*p<0.05) using GraphPad Prism

The three additional parameters of PA, HR, and T_CO_ all behaved differently compared to mean arterial pressure (Fig. 3). PA was generally not altered as a result of the O_3_ exposure (Fig. 3A). Activity was elevated during pairing with male rats, and reduced for approximately 24h following the O_3_ or sham exposure. Moreover, there was a steady reduction in PA throughout the course of pregnancy. Even with removing the first 4 days of data to eliminate effects of pairing with male rats, Time as a factor in the 2-way repeated measures ANOVA was significant (p = 0.0013) while O_3_ was not.

HR was acutely reduced by the O_3_ exposure, on average 43 beats per minute lower than FA-exposed dams and fully resolving 48–72h post exposure (Fig. 3B). During the period of elevated mean arterial pressure (GD15.5–GD19.5), however, HR values were similar between exposure groups. This acute reduction in HR is consistent with numerous previous studies [15, 16]. Interestingly, HR increased in both groups peridelivery, despite no increase in PA.

T_CO_ was consistent between both groups prior to exposure, but displayed an interesting reduction late in pregnancy, significant on GD17.5 (Fig. 3C). This aligns well with the general period of elevated mean arterial blood pressure (Fig. 2B and C). Previous research has routinely described an acute hypothermia in mice and rats associated with O_3_ exposure [16], but this delayed response appears unique to the pregnant rat. This acute phenomenon of hypothermia could be observed in the present study if data was viewed more closely (Fig. 3D). The drop in T_CO_ was substantial (mean = 33.2°C), but resolved within approximately 2h after the O_3_ exposure ended.

## Discussion

The use of radiotelemetry in a Sprague Dawley rat model of pregnancy has been employed in several studies to evaluate changes in blood pressure, ostensibly to mimic gestational hypertension and/or preeclampsia. Infusion of the vasoconstrictor angiotensin II on gestational days 6, 9, 12, and 15 has been used to increase mean arterial blood pressure about 20 + mmHg over controls, while maintaining the integrity of the pregnancy [17]. Severe hypertensive disorders of pregnancy, such as preeclampsia, have also been modeled utilizing intraperitoneal injection of the angiogenesis inhibitor Suramin to increase (17 mmHg) blood pressure [18]. To our knowledge, this is the first study utilizing radiotelemetry to continuously assess blood pressure of unrestrained pregnant rats in response to an inhaled toxicant such as ozone. Previous studies have examined blood pressure intermittently following ozone exposure in pregnant rats via tail cuff photoplethysmograpy and saw no blood pressure changes [7], although slight differences in O_3_ concentration as well as exposure timing complicate comparing the two studies.

O_3_ exposure occurring during the initial phases of placentation (GD10.5) led to a delayed elevation of blood pressure in pregnant rats several days following the exposure. While the effect was mild (only a 5 mmHg increase), it was sustained until delivery, consistent with human gestational hypertension, i.e., elevated blood pressure of placental origin. The blood pressure changes were accompanied by a modest reduction in T_CO_, which may indicate a general morbidity associated with the late stage condition.

Changes acutely caused by O_3_ exposure, namely bradycardia and hypothermia, are typical of such exposures and rapidly resolved, as previously reported.[15, 16, 19] For reasons described below, the delayed pressor effect of O_3_ in the present study is likely a unique pathology elicited by indirect effects on placental growth, setting the stage for a syndrome akin to gestational hypertension in late stages of pregnancy.

Mothers are highly vulnerable to pathological cardiovascular and hemodynamic alterations during pregnancy, as the growing fetus and palcenta demand a substantive increase in cardiopulmonary output over the 9 months of gestation [20]. Under normal circumstances, maternal vascular resistance drops approximately 30% to accommodate increased demands on cardiac output from the growing fetus and the typical mild anemia that occurs. However, environmental factors may interfere with the normal vascular remodeling required for this reduction in systemic resistance, promoting hypertension and cardiomyopathy.

Placental insufficiency is linked to maternal hypertension and preeclampsia. Preeclampsia occurs in roughly 3–8% of pregnancies, reflecting a large population at risk, but numerous factors contribute to the etiology.[21] Incomplete angiogenesis during placentation can lead to a poorly perfused placenta, which responds by releasing proangiogenic and proinflammatory factors.[22] Maternal systemic blood pressure surges dramatically in preeclampsia, accompanied by proteinuria and elevations in several key circulating factors such as the soluble fms-like tyrosine kinase-1.[23] In addition to acute risk of seizure, death, or pre-term delivery resulting from a hypertensive crisis, long-term cardiomyopathic, hemodynamic, and cognitive deficits have been observed in preeclampsia patients post-delivery.[24, 25] Preeclampsia incidence has a heterogeneous etiology and is influenced in part by genetics, immunology, nutritional status, and environmental exposures,[26–30] but no single factor has a strong predictive role. The present study and other recent research may provide an integrated mechanistic understanding of how air pollution may adversely impact placental development, promoting preeclampsia and related maternal cardiovascular pathologies.

**Figure 4:**
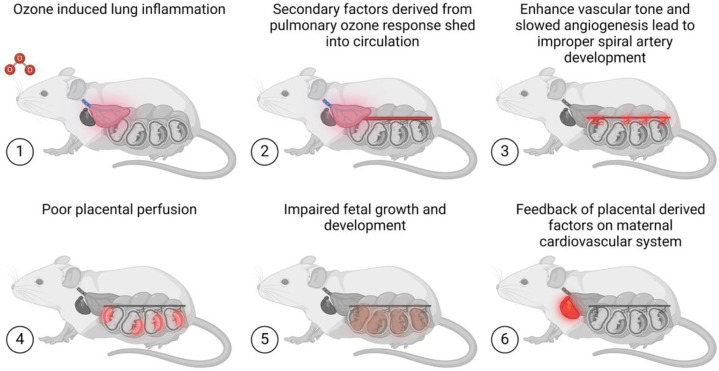
Summarizing studies from our laboratory and others, we propose that inhaled pollutants such as ozone can promote or exacerbate gestational by causing circulating factors arising from the lung that retard placental angiogenesis, which ultimately leads to placental insufficiency later in pregnancy. Figure created using Biorender.

As an important component of the mechanisms by which inhaled pollutants impact the systemic vasculature, we have identified that fragmented peptides arising from pulmonary peptidase activity in response to pollutants [31] can directly stimulate endothelial inflammatory responses in coronary, mesenteric, and cerebrovascular beds [32–34], and there is little reason to assume that the placental vascular bed is not similarly vulnerable to the systemic effects of inhaled toxicants. As a specific example, a fragmented thrombospondin-1 peptide (corresponding to amino acids 402–460) was highly upregulated in serum after pulmonary particulate matter exposure and was observed to reduce endothelial cell growth rates, most likely via interaction with the CD36 receptor.[34, 35] We postulate that such mechanisms may directly retard early placental growth especially of the vasculature. Later in gestation, the delayed placental growth may lead to release of pressors like sFlt-1 to direct greater blood flow to the placenta, elevating maternal vascular resistance and blood pressure (Fig. 4).

One limitation to this study design is that blood pressure response to O_3_ was not assessed in non-pregnant rats. Notably several studies have investigated this with limited indication that blood pressure or hemodynamics, in general, are impacted by O_3_. Numerous studies even point to a reduction in blood pressure in vulnerable or two-hit models designed to evince hypertension.[36–40] Normally, O_3_ is rapidly scavenged in the epithelial lining fluid of the lung and inflammatory/toxic effects are typically acute and resolved within 24–72 hours, depending on the exposure concentration and extent of lung injury.[41–43] The concentration used in the present study (1 ppm) typically resolves in 24h. Thus, the observation of significant mean arterial pressure changes, along with T_CO_ changes, arising 5–10 days after the exposure suggests there is a unique response in the setting of pregnancy that may promote the pathology of gestational hypertension. Early O_3_-induced deficits of placental growth and development might therefore set the stage for increased uterine artery resistance and ultimately higher blood pressure as demands of the fetus and placenta increase in late pregnancy.

A second limitation to this study is the rat model where the differences in gestational timing, litter size, and possibly placental physiology may limit translation to human gestation. However, the differences may also explain why the gestational hypertension seen in the present study was only a modest 5 mmHg, while human preeclampsia is characterized blood pressures exceeding 200 mmHg, *i.e*., to life-threatening levels. The perfusion of the rodent uterus may become limited by the O_3_ effect on uterine vascular resistance, but the rodent can reabsorb fetuses to balance this outcome and ensure a healthy supply of blood to all progeny, which humans are unable to do. Furthermore, the short duration of gestation (22 days) may simply not be sufficient to generate a profound hypertensive phenotype. Thus, we suspect the mild cardiovascular response in the rodent model implies that increasing the severity of O_3_ exposure would lead to spontaneous abortion.

In summary, pregnant rats exposed to O_3_ at GD10.5 displayed a mild but significant increase in mean arterial blood pressure beginning 5 days after exposure and lasting until term. While this model has limitations and the scope of pathological assessment does not permit a stronger conclusion related to gestational hypertension or preeclampsia, the phenomenon of late-term elevated blood pressure that resolves after delivery is consistent with the debilitating and life-threatening human syndrome of preeclampsia. Integrated within the context of several recent research communications, this adds meaningfully to the understanding of how ambient air pollution may promote the risk of adverse pregnancy outcomes like gestational hypertension or spontaneous abortion. Much research is needed to better delineate the link between pulmonary exposures and placental effects as well as the relative contribution of summated pollutants, diet, genetics and lifestyle that may further contribute to risk of adverse outcomes.
